# Associations of ethylene oxide exposure with depression in American adults

**DOI:** 10.1038/s41598-024-64908-6

**Published:** 2024-06-24

**Authors:** Meng Wang, Chao Liu, Quan Liu, Ruizhen Bai

**Affiliations:** 1 Department of Oncology, Affiliated Hospital of Jiangnan University, Wuxi, 214122 China; 2 Department of Epidemiology and Statistics, Bengbu Medical University, Bengbu, China; 3 Department of Pathology, Affiliated Hospital of Jiangnan University, Wuxi, 214122 China

**Keywords:** Environmental impact, Depression

## Abstract

Ethylene oxide (EO) is an organic compound known for its high reactivity and negative impact on human health, but its adverse effects on depression remain poorly understood. A cross-sectional study was conducted among 2884 participants from the National Health and Nutrition Examination Survey (NHANES) between 2013 and 2016. Participants were classified into four groups according to quartiles of log10-transformed hemoglobin adducts of EO (HbEO) levels. A logistic regression model was used to estimate the association between EO exposure and the risk of depression. Finally, we evaluated whether the association was mediated by inflammatory factors. Individuals with depression exhibited higher levels of hemoglobin adducts of ethylene oxide (HbEO) compared to those without depression. After adjusting for all covariates, patients in the highest quartile of HbEO (Q4 group) had a higher risk of depression, using the lowest quartile (Q1 group) as the reference group [odds ratio (*OR*) = 2.21, 95% confidence interval (95% CI): (1.47, 3.40)]. Additionally, the relationship between EO levels and the prevalence of depression followed a non-linear U-shaped pattern. Furthermore, inflammatory cells showed a positive correlation with EO levels. Moreover, white blood cells and neutrophils significantly mediated the relationship between HbEO and the risk of depression with mediated proportions of 14.70 and 12.55%, respectively. Exposure to ethylene oxide increases the risk of depression. Inflammatory factors partially mediated the observed association between EO exposure and depression.

## Introduction

Depression is a prevalent chronic medical condition that can impact an individual’s cognition, mood, and overall physical well-being. It is characterized by persistent low mood, a noticeable decrease in energy levels, sadness, difficulties in sleeping, and a diminished capacity to find joy in life’s experiences^1^. Depression contributes to a large global burden of disease, with the World Health Organization (WHO) ranking major depression as the third leading cause of the global burden of disease in 2008 and predicting that it will progress to first place by 2030^2,3^. According to the data of WHO, the global prevalence of depression stands at approximately 300 million people, with the number of cases steadily rising each year^4^. Although depression can be treated with medications, psychosocial interventions, and brain stimulation, it remains a considerable challenge to effectively manage this condition^5^. Unfortunately, more than three-quarters of individuals with mental disorders in low- and lower-middle-income countries do not have access to treatment^6^, highlighting the need for innovative preventive measures. While various factors contribute to depression, emerging research suggests that environmental factors may play a crucial role. For instance, exposure to environmental toxins poses a severe threat to global public health.

Ethylene oxide (EO) is a toxic industrial chemical used in medical device sterilization, plastics and textile manufacturing, and other industrial applications^7^. It forms hemoglobin adducts of EO (HbEO) by binding with hemoglobin (Hb), which serves as a specificity indicator to assess ethylene oxide exposure^8^. EO is present in the gaseous state at room temperature, with inhalation being the primary route of exposure. The general population is primarily exposed to EO through contaminated air, cigarette smoke, and vehicle exhaust fumes^9^. Recent studies have provided substantial evidence linking ethylene oxide exposure to detrimental effects on human health. As a component of cigarette smoke, EO is recognized as a potential exogenous toxicant with adverse effects on human health^10^. Jinot et al. found that EO can combine with proteins and nucleic acids to form macromolecular compounds, thus some undesirable human hazards^11^. In addition, some evidence suggests that inhalation and dermal exposure to ethylene oxide can have an impact on the development of a number of chronic diseases, such as an increased risk of diabetes, hypertension, cardiovascular disease, asthma, and kidney stones^9,12,13^. Regarding possible mechanisms, there are indications that inflammation may play an important role in the link between EO and diseases (e.g., chronic obstructive pulmonary disease, asthma, diabetes, kidney stones, etc.)^9,12,14,15^. However, the potential adverse effects of EO exposure on depression remain poorly understood. Considering the increasing number of people currently suffering from depression and the adverse health effects of ethylene oxide, there is a need to explore the relationship between EO exposure and depression in order to further inform the treatment of depression and the use of EO.

In summary, we investigated the association between EO exposure and depression by using the data from the National Health and Nutrition Examination Survey (NHANES). In order to further elucidate the potential pathogenesis, we also explored the mediating role of inflammation markers between EO and depression.

## Methods

### Study population

A nationally representative cross-sectional study, the National Health and Nutrition Examination study (NHANES) gathers information on the eating habits, nutritional status, socioeconomic status, and demographics of the civilians in the United States^16^. Each participant signed an informed consent form in compliance with the protocol authorized by the ethics review board of the US National Center for Health Statistics (NCHS). The data, methodology, and design of the survey are all openly accessible (https://www.cdc.gov/nchs/nhanes/about nhanes.htm).

**Figure 1:**
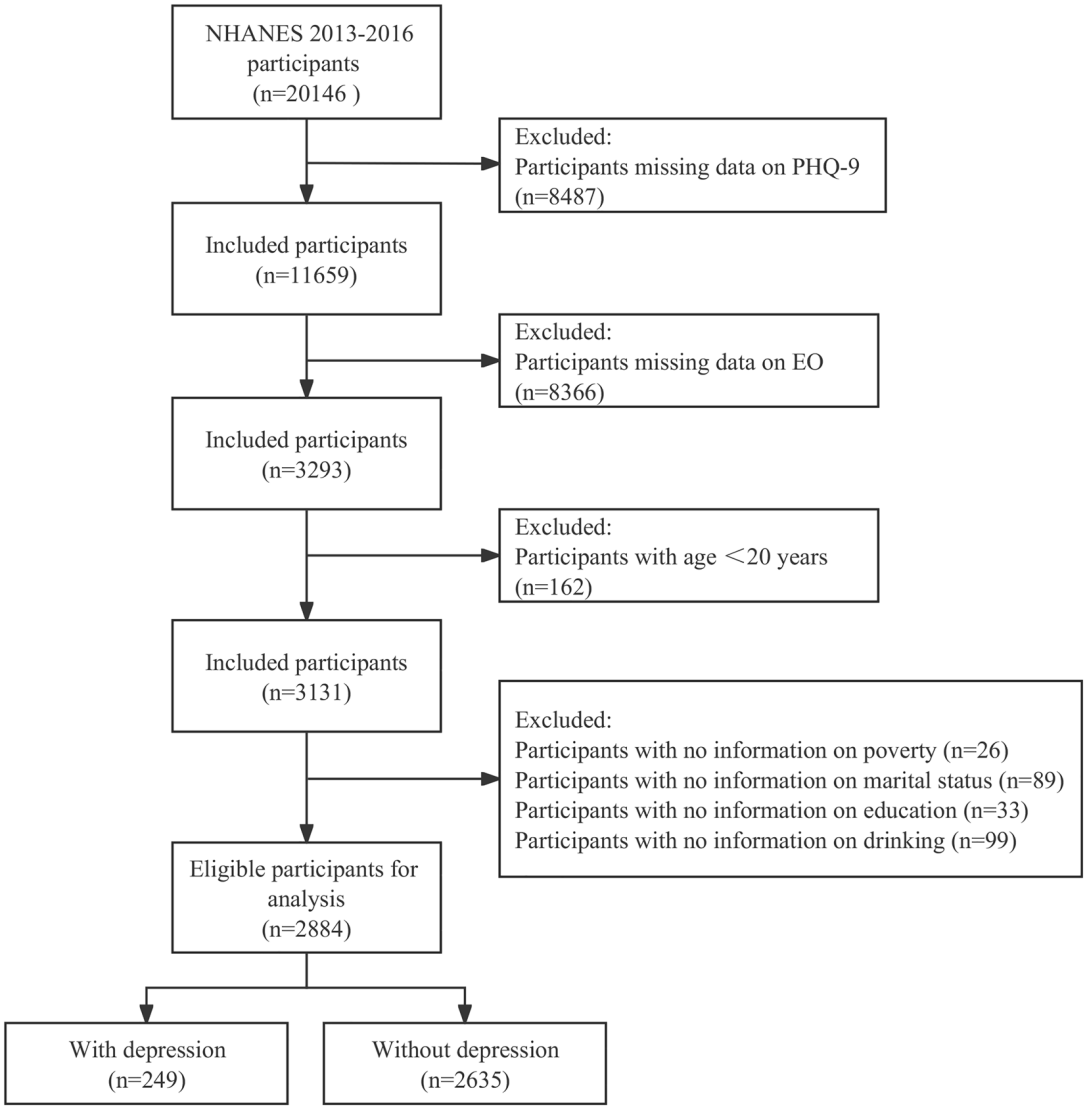
Flowchart of participants selection from NHANES 2013–2016.

Among 20,146 participants in the NHANES 2013–2016, individuals were excluded if (1) they had incomplete PHQ-9 data (n = 8487), (2) they lost EO exposure data (n = 8366), (3) they were less than 20 years old (n = 162), (4) they had missing data on covariates data included poverty (n = 26), marital (n = 89), education (n = 33), and drinking (n = 99). In our final analysis, 2884 eligible participants who were at least 20 years old were included. (Fig. 1).

### Measurement of EO

This study used HbEO as a measure of because it has been shown to be a highly sensitive method for determining ethylene oxide exposure^17^. The procedure for measuring HbEO can be accessed at: https://wwwn.cdc.gov/Nchs/Nhanes/2015-2016/ETHOX_I.htm. The above methods have been described in detail by several studies^12,18,19^. In short, the red blood cell sample underwent a series of processes including washing, packaging, processing, and shipping to the Division of Laboratory Sciences at the National Center for Environmental Health. Following storage at − 30 °C, the sample was then shipped to the National Center for Environmental Health for evaluation. The total hemoglobin level was measured and identified, and the estimated hemoglobin adducts were determined. The modified Edman reaction was used to determine Hb levels, utilizing the commercial assay kit from Tech Diagnostics in Anaheim, CA. Additionally, the HbEO level in both human whole blood and erythrocytes was determined through high-performance liquid chromatography coupled with tandem mass spectrometry (HPLC–MS/MS). Additional experimental details are available in the NHANES Laboratory/Medical Technician Procedures Manual.

### Assessment of depression

To diagnose depression, the Patient Health Questionnaire (PHQ-9) was utilized. This self-reported assessment is based on the nine signs and symptoms of depression in the Diagnostic and Statistical Manual of Mental Disorders, Fourth Edition (DSM-IV)^20^. Using this instrument, depressive symptoms can be assessed for the past two weeks^21^. It consists of nine questions. Each query is assessed on a scale of 0 (not at all) to 3 (nearly daily), accumulating a total score ranging from 0 to 27. An aggregated score exceeding 10 is deemed to reflect the presence of depressive symptoms, or alternatively, it classifies individuals into a non-depressed category^22^. The magnitude of depressive symptoms is directly proportional to the PHQ-9 score, with higher scores indicating more severe symptoms. According to the score, the patients could be divided into depression group and non-depression group. Previous depressive episodes in the studied population are variables influencing depressive episodes. In this study, grouping was based only on PHQ-9 scores. Participants with a previous depressive episode score ≥ 10 were considered to have depression. If the score was less than 10, they were classified as non-depressed.

### Covariates

The following covariates were incorporated into our analysis: (1) demographic factors: age, sex, ethnicity, educational attainment, marital status, and household poverty-to-income ratio (PIR); (2) examination data: body mass index (BMI); (3) questionnaire data: hypertension, and alcohol consumption; (4) laboratory test results: white blood cell, lymphocytes, monocytes, and neutrophils levels. These variables in NHANES website has described, which can be visited at https://wwwn.cdc.gov/Nchs/Nhanes/continuousnhanes. We specifically categorized the above covariates based on NHANES criteria and previous studies^14,18,23^. The racial categories included Mexican American, Non-Hispanic Black, Non-Hispanic White, Other Hispanic, and other races. Educational attainment was categorized into three groups: middle school or lower, high school, and above high school. Marital status was classified according to the NHANES criteria: married, never married, and other. PIR was estimated by dividing household income by the poverty line, with participants classified as low-income (PIR < 1.3), middle-income (PIR = 1.3–3.50), and high-income (PIR ≥ 3.50)^14^. Body mass index (BMI) was calculated by dividing weight (kg) by height (m) squared, and was categorized as < 25 kg/m^2^ and ≥ 25 kg/m^2^. Hypertension was based on self-reported hypertension. The four inflammatory factors included in the laboratory parameters were extracted from whole blood cells (Laboratory data: CBC H Data). The methods used to derive CBC parameters are based on the Beckman Coulter method of counting and sizing, in combination with an automatic diluting and mixing device for sample processing, and a single beam photometer for hemoglobinometry. The whole blood count (WBC) differential uses VCS technology. See Chapter 7 of the NHANES Laboratory Procedures Manual (LPM) for details. The Beckman Coulter D × H 800 instrument in the NHANES mobile examination center (MEC) produces a complete blood count on blood specimens and provides a distribution of blood cells for all participants. Refer to the Laboratory Method Files section for detailed laboratory procedure manual(s) of the methods used.

### Statistical analysis

Based on baseline characteristics, the participants were classified into two groups based on their depression status. Continuous variables were expressed as means ± standard deviations and subjected to *t*-test analysis. HbEO levels were log10-transformed to normalize the distributions and divided it into quartiles^12,14^. Univariate and multivariate logistic regression models were used to evaluate the odds ratios (ORs) and 95% CIs of the associations between log10-transformed HbEO and the risk of depression. We further classified log10-transformed HbEO into quartiles: Quartile 1 (lowest), Quartile 2, Quartile 3, and Quartile 4 (highest). We calculated the change (OR and 95CI%) in the risk of developing depression in each quartile relative to the lowest quartile (Quartile 1). Then,by including the median values of the HbEO quartiles as a continuous variable in the models, we looked for a tendency related to the risks.

In this study, we constructed three models: crude model did not consider any covariates; Model Icontained only age and sex; Model IIcontained all covariates. We explored the shape of the non-linear exposure–response relationships of log10-transformed HbEO with the risk of depression using restricted cubic spline (RCS) with three knots. Stratified analyses were performed by gender (male vs. female), age (< 60 years vs. ≥ 60 years), BMI(< 25 kg/m^2^ vs. ≥ 25 kg/m^2^), Hypertension (yes vs. no), Education (middle school or lower vs high school), Drinking (yes vs. no), PIR (< 1.3 vs. 1.3–3.5 vs. > 3.5), Marital status (never married vs. married vs. others), and Ethnicity. Then,we tested effect modification^24,25^ in subgroup analyses and considered the effect to be present if a regression cross-product term (e.g., HbEO × age) was statistically significant. Furthermore, we further explored the association between HbEO levels and indicators of inflammation by using multivariate linear regression model. Finally, we assessed the potential mediating effects of inflammatory factors on EO and depression. All data analyses were performed using R (version 4.0). *P* < 0.05 was considered statistically significant.

## Results

### Participant characteristics

A total of 2884 subjects (1429 males and 1455 females), with an average age of 49.15 ± 17.47 years were enrolled (Table 1). Compared to the participants without depression (n = 2635), individuals with depression (n = 249) exhibited a higher likelihood of being female, having lower levels of education and income in relation to poverty, engaging less frequently in alcohol consumption, and experiencing a greater prevalence of hypertension.

**Table 1:** Characteristics of participants from NHANES 2013–2016.

Characteristics	Total participants	Depression situation		*P*-value
		No (PHQ < 10)	Yes (PHQ ≥ 10)	
All participants	2884	2635	249	
Age, year	49.15 ± 17.47	49.05 ± 17.54	50.22 ± 16.64	0.291
Gender				0.002
Male	1429 (49.55%)	1329 (50.44%)	100 (40.16%)	
Female	1455 (50.45%)	1306 (49.56%)	149 (59.84%)	
Ethnicity				0.125
Mexican America	428 (14.84%)	397 (15.07%)	31 (12.45%)	
Non-Hispanic Black	301 (10.44%)	271 (10.28%)	30 (12.05%)	
Non-Hispanic White	1192 (41.33%)	1081 (41.02%)	111 (44.58%)	
Other Hispanic	564 (19.56%)	510 (19.35%)	54 (21.69%)	
Other	399 (13.83%)	376 (14.27%)	23 (9.24%)	
Education				< 0.001
Middle school or lower	593 (20.6%)	512 (19.43%)	81 (32.53%)	
High school	646 (22.4%)	581 (22.05%)	65 (26.10%)	
Above high school	1645 (57.0%)	1542 (58.52%)	103 (41.37%)	
Marital status				< 0.001
Never married	556 (19.28%)	503 (19.09%)	53 (21.29%)	
Married	1455 (50.45%)	1367 (51.88%)	88 (35.34%)	
Other	873 (30.27%)	765 (29.03%)	108 (43.37%)	
PIR				< 0.001
< 1.3	930 (32.25%)	791 (30.02%)	139 (55.82%)	
1.3–3.5	1080 (37.45%)	999 (37.91%)	81 (32.53%)	
> 3.5	874 (30.31%)	845 (32.07%)	29 (11.65%)	
BMI(kg/m^2^)				0.114
< 25	783 (27.15%)	726 (27.55%)	57 (22.89%)	
≥ 25	2101 (72.85%)	1909 (72.45%)	192 (77.11%)	
Drinking				< 0.001
No	464 (16.09%)	403 (15.29%)	61 (24.50%)	
Yes	2420 (83.91%)	2232 (84.71%)	188 (75.50%)	
Hypertension				< 0.001
No	1839 (63.77%)	1718 (65.20%)	121 (48.59%)	
Yes	1045 (36.23%)	917 (34.80%)	128 (51.41%)	
EO (pmol/g Hb)	62.29 ± 106.44	58.31 ± 102.74	104.39 ± 132.81	< 0.001
LogEO	1.48 ± 0.46	1.46 ± 0.44	1.67 ± 0.56	< 0.001

*PIR*, family poverty income ratio; *BMI*, body mass index; *EO*, ethylene oxide; *LogEO*, log10-transformed ethylene oxide. *P* < 0.05 was considered a statistically significant difference between the depressed and non-depressed groups for the different characteristic groups.

### Association of EO and depression

As presented in Table 2, HbEO and the risk of depression showed a significant connection, according to multivariate logistic regression analysis (*OR*: 2.42, 95% CI: 1.88, 3.10). Confounding factor adjustments were made for model I (*OR*: 2.63, 95% CI: 2.04, 3.39) and model II (*OR*: 1.90, 95% CI: 1.43, 2.51).

**Table 2:** Multivariate logistic regression analysis of log10-transformed HbEO for prevalence of depression.

	Crude model		Model I		Model II	
	Crude OR (95% CI)	*P*-value	Adjusted OR (95% CI)	*P*-value	Adjusted OR (95% CI)	*P*-value
Log10-HbEO	2.42 (1.88, 3.10)	< 0.001	2.63 (2.04, 3.39)	< 0.001	1.90 (1.43, 2.51)	< 0.001
Q1	Ref		Ref		Ref	
Q2	1.10 (0.71, 1.73)	0.678	1.10 (0.70, 1.72)	0.690	1.12 (0.71, 1.78)	0.627
Q3	1.10 (0.71, 1.73)	0.668	1.12 (0.72, 1.77)	0.611	1.11 (0.70, 1.78)	0.649
Q4	2.87 (1.97, 4.29)	< 0.001	3.17 (2.15, 4.75)	< 0.001	2.21 (1.47, 3.40)	< 0.001
*P* for trend	< 0.001		< 0.001		< 0.001	

The crude model was not adjusted for covariates. Model Iwas adjusted for age, gender. Model IIwas adjusted for all covariates. *HbEO*, hemoglobin adducts of ethylene oxide; *OR*, odd ratio; *CI*, confidence interval. *P* for trend < 0.05 indicated that the trend test is statistically significant. *P*-value < 0.05 indicated a statistically significant effect of ethylene oxide exposure on the presence or absence of depression.

To further analyze the data, we further divided the log10-transformed HbEO into 4 quartiles, with quartile 1 being the lowest exposure. For each of the three models, the quartile 4 showed a significantly higher prevalence of depression than the quartile 1 (quartile 4 in crude model = *OR*: 2.87, 95% CI: 1.97, 4.29, *P* for trend < 0.001; quartile 4 in model I: *OR* = 3.17, 95% CI: 2.15, 4.75, *P* for trend < 0.001; quartile 4 in model II: *OR* = 2.21, 95% CI: 1.47, 3.40, *P* for trend < 0.001).

**Figure 2:**
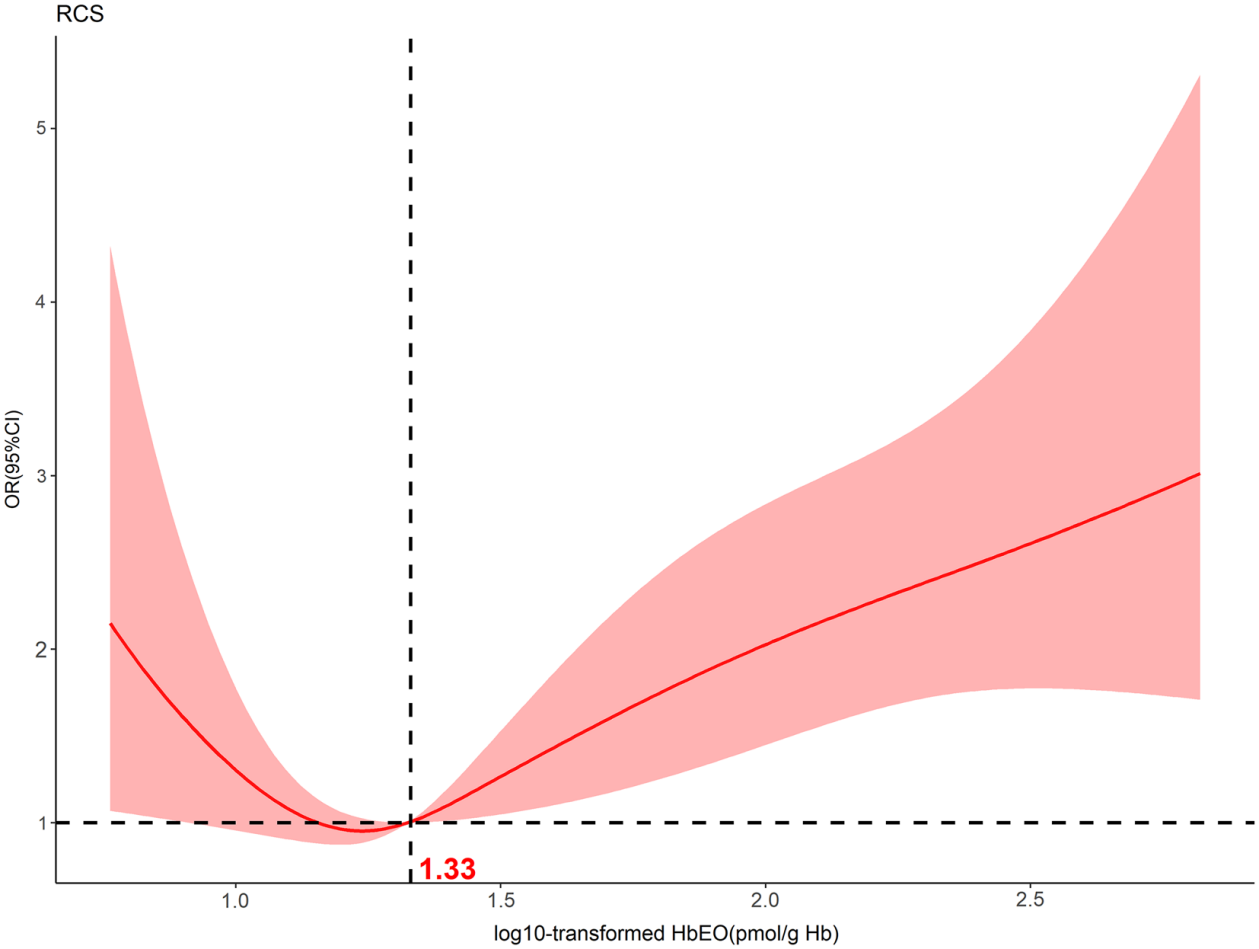
Relative risk of depression according to log10-transformed hemoglobin ethylene oxide (HbEO) levels in the overall population. The solid line and shadow represented the odds ratio of depression and 95% confidence interval, respectively. Dashed vertical line indicated the threshold (log10-transformed HbEO = 1.33 pmol/g Hb) with the lowest risk of depression. All covariates were adjusted in this model.

Furthermore, the association between HbEO levels and the prevalence of depression displayed a significant non-linear U-shaped pattern even after accounting for potential confounding factors. After reaching a low point of 1.33 pmol/g Hb for log10-transformed HbEO, the incidence of depression began to rise (Fig. 2).

### Subgroup analysis

To assess the consistency of the relationship between depression prevalence and log10-transformed HbEO levels across different categories, subgroup analysis was conducted (Table 3). Significant correlations were observed in each subgroup (all *P* < 0.05) when stratified by age, gender, BMI, hypertension, education, drinking habits, ethnicity, and marital status. However, our further interaction tests disclosed that no statistically significant interactions were observed in above stratification (*P* for interaction > 0.05), except PIR. The resluts showed that factors such age, gender, BMI, hypertension, education, drinking habits, ethnicity, and marital status did not influence the association between HbEO levels and the risk of depression. Among participants with a PIR ≤ 3.50, there was a statistically significant association between HbEO and depression (*P* < 0.05). Although a positive association between HbEO and depression was also found in participants with a PIR > 3.50, it did not reach statistical significance (*OR*: 1.11, 95% CI: 0.72, 1.66, *P* = 0.639). Given the significant interaction between blood ethylene oxide levels and PIR groups identified in our study, further analysis based on PIR strata indicated that the substantial association of blood ethylene oxide levels with depression prevalence was primarily evident among participants with a PIR ≤ 3.50 rather than those with a higher income level (PIR > 3.50) (*P* for interaction = 0.033).

**Table 3:** Subgroup analyses on the association between blood HbEO levels and depression.

Characteristics	OR (95% CI)	*P*-value	*P* for interaction
Age (years)			0.224
< 60	1.93 (1.47, 2.54)	< 0.001	
≥ 60	1.58 (1.12, 2.22)	0.009	
Gender			0.110
Male	1.52 (1.14, 2.03)	0.005	
Female	2.13 (1.59, 2.85)	< 0.001	
BMI(kg/m^2^)			0.721
< 25	1.69 (1.26, 2.25)	< 0.001	
≥ 25	1.88 (1.41, 2.52)	< 0.001	
Hypertension			0.637
No	1.56 (1.15, 2.11)	0.004	
Yes	2.19 (1.64, 2.92)	< 0.001	
Education			0.857
Middle school or lower	2.17 (1.62, 2.90)	< 0.001	
High school	1.98 (1.49, 2.62)	< 0.001	
Above high school	1.71 (1.25, 2.33)	< 0.001	
Drinking			0.931
No	2.10 (1.54, 2.86)	< 0.001	
Yes	1.77 (1.32, 2.35)	< 0.001	
PIR			0.033
< 1.3	2.19 (1.65, 2.90)	< 0.001	
1.3–3.5	1.73 (1.26, 2.35)	< 0.001	
> 3.5	1.11 (0.72, 1.66)	0.639	
Ethnicity			0.324
Mexican America	1.60 (1.07, 2.37)	0.021	
Non-Hispanic Black	1.88 (1.26, 2.77)	0.002	
Non-Hispanic White	1.98 (1.49, 2.63)	< 0.001	
Other Hispanic	1.71 (1.25, 2.31)	< 0.001	
Other	1.73 (1.16, 2.54)	0.006	
Marital status			0.616
Never married	1.99 (1.43, 2.75)	< 0.001	
Married	1.64 (1.19, 2.24)	0.002	
Other	1.95 (1.46, 2.59)	< 0.001	

*HbEO*, hemoglobin adducts of ethylene oxide; *PIR*, family poverty income ratio; *BMI*, body mass index; *OR*, odd ratio; *CI*, confidence interval. *P*-value < 0.05 indicated a statistically significant effect of ethylene oxide exposure on depression in each subgroup. *P* for interaction < 0.05 indicated that the difference in the interaction term is statistically significant.

### Association of inflammation parameters and HbEO

Utilizing a multivariate linear regression model, the relationship between inflammatory variables and HbEO was examined. Table 4 shows that HbEO had a significantly positive association with white blood cells (*β*: 1.10, 95% CI: 0.92, 1.28), lymphocyte cells (*β*: 0.28, 95% CI: 0.22, 0.34), monocyte cells (*β*: 0.04, 95% CI: 0.03, 0.06), neutrophils cells (*β*: 0.76, 95% CI: 0.61, 0.90). These findings suggested that HbEO was connected to inflammation.

**Table 4:** Multivariate linear regression of log10-transformed HbEO levels with inflammatory factors.

Inflammatory indicators	n	β	95% CI	*P*-value
White blood cell (1000 cells/μL)	2873	1.10	0.92, 1.28	< 0.001
Lymphocyte cell (1000 cells/μL)	2873	0.28	0.22, 0.34	< 0.001
Monocyte cell (1000 cells/μL)	2873	0.04	0.03, 0.06	< 0.001
Neutrophils cell (1000 cells/μL)	2873	0.76	0.61, 0.90	< 0.001

All covariates were adjusted in this model. *HbEO*, hemoglobin adducts of ethylene oxide; *CI*, confidence interval. *P*-value < 0.05 indicated a linear and statistically significant relationship between the levels of inflammatory factors and ethylene oxide.

### Inflammation parameters involved in the effects of HbEO on depression

As shown in Table 5, we further explored the mediating role of inflammatory factors on the association between HbEO and depression by mediation analysis. White blood cells and neutrophil cells were found to have a significant mediating effect on the association between HbEO and depression, with mediation ratios of 14.70% (*P* < 0.001) and 12.75% (*P* = 0.002), respectively. However, no significant mediating effect was found for lymphocyte cells and monocyte cells (*P* > 0.05).

**Table 5:** The mediating effects of inflammatory factors on the association between log10-transformed HbEO and depression.

Inflammatory factors	Indirect effects	Direct effects	Total effects	Mediated (%)	*P*-value
	β (95% CI)	β (95% CI)	β (95% CI)		
White blood cell	0.004 (0.001, 0.01)	0.023 (0.010, 0.03)	0.027 (0.013, 0.03)	14.70	< 0.001
Lymphocyte cell	0.000 (− 0.002, 0.00)	0.028 (0.017, 0.03)	0.028 (0.019, 0.03)	0.95	0.72
Monocyte cell	0.001 (− 0.001, 0.00)	0.027 (0.019, 0.03)	0.028 (0.019, 0.03)	1.64	0.52
Neutrophils cell	0.004 (0.001, 0.01)	0.024 (0.014, 0.03)	0.027 (0.019, 0.03)	12.75	0.002

All covariates were adjusted in this model. *HbEO*, hemoglobin adducts of ethylene oxide; *CI*, confidence interval; *P*-value < 0.05 indicated statistically significant mediation of inflammatory factors between EO and depression.

## Discussion

As far as the authors are aware, this is the first research to examine the relationship between the prevalence of depression and the EO exposure. Our findings indicated that ethylene oxide exposure was significantly correlated with depression, characterized by a U-shaped curve. In addition, we also found that white blood cells and neutrophil cells partially mediated these associations. Potential findings from this investigation might be useful in understanding the relationship between EO exposure and depression. These findings could open up new possibilities for addressing the detrimental impact of EO on depression and raising awareness of the illness’s prevalence.

As a volatile organic, EO has been recognized as posing a major threat on human health^12,14,18,26^. For instance, ethylene oxide has been internationally recognized as a human carcinogen^26^. There is evidence that links EO exposure to the development of dyslipidemia, hypertension, and diabetes mellitus^27,28^. In addition, several studies^29–32^ have shown that ethylene oxide exposure is associated with cognitive function, neurological dysfunction. A United States (US) clinical study^4^ found that psychomotor abilities and nerve conduction velocities were reduced in people with prolonged exposure to ethylene oxide. Another U.S. clinical study^31^ found the after ethylene oxide exposure, subjects have a statistically significant reduced P300 amplitude, diminished bilateral distal deep tendon reflexes and performed poorly on neuropsychological tests involving psychomotor speed. Furthermore, Patch et al. found when exposed to ethylene oxide for a prolonged period of time, people’s mental abilities were affected and they also developed anxiety^32^. Nevertheless, no research has examined the connection between depression and EO exposure. A U-shaped relationship between EO exposure and depression was discovered in this investigation, and when log10-transformed HbEO was greater than 1.33 pmol/g Hb, the risk of depression was considerably higher. These findings suggest that exposure to EO may have a role in the etiology of depression.

Although the underlying mechanisms of the negative effect of EO exposure on the depression are not yet clarified, several explanations have been suggested. For example, the relationship between ethylene oxide and inflammatory factors has been widely reported. According to Zeng et al., exposure to EO may impact fatty acid metabolism and inflammatory responses, which may lead to the development of cardiovascular disease^18^. According to LIet al., a plausible molecular explanation for EO-induced asthma might be the inflammatory response^3^. Huang et al. found that one important mediator in the effects of EO exposure on chronic obstructive pulmonary disease (COPD) is inflammation^2^. Reduced intracellular glutathione levels and increased hepatic lipid peroxidation, both of which are linked to oxidative stress in vivo, were found to be caused by EO exposure in a number of earlier experimental animal studies^29,33,34^. Notably, a large body of research has shown that inflammation, immunity, and oxidative stress all have a significant part in depression^35,36^. Our findings supported earlier research by showing a favorable relationship between HbEO and inflammatory variables, which in turn mediated HbEO’s effects on depression. Consequently, it makes sense to hypothesize that inflammation might be a possible mechanism behind depression linked to EO^37^.

We further performed subgroup analyses to verify the stability of above findings and the result revealed that no statistically significant interaction was observed in subgroup when stratified by age, gender, BMI, hypertension, education, drinking habits, ethnicity, and marital status. Similar to the current study, in the research about EO-realted hazards, one investigation revealed that stratification variables, including age,gender,and BMI, drinking, had no interactive effect on the correlation between EO and the risk of diabetes^9^. And another study also exhibited that there was no interaction on the association of HbEO with albuminuria in terms of gender, BMI, hypertension and other factors^38^. These resluts showed that age, gender, BMI, and other factors had no interaction effect on the association between HbEO levels and depression prevalence. But, we found that the association between EO exposure and depression differed significantly (*P* for interaction = 0.033) by family income. The effect of EO exposure on depression was not statistically significant (*P* = 0.639) when the PIR was greater than 3.5. This may be because people with high household incomes have better living conditions and psychological status.

Our research has several advantages. This study was the first to investigate the connection between blood EO exposure and the overall depression prevalence in the United States population. In order to get more trustworthy findings, we took confounding factors into account. The association between HbEO levels and depression was examined, and systemic inflammation was further demonstrated by mediation analysis as a potential cause. There are a few things to consider regarding this study’s limitations. First, because of the study’s cross-sectional design, we were unable to demonstrate a causal relationship between HbEO and depression and the mediation effects of inflammatory factors, which needs to be further validated in prospective cohort studies. Secondly, our results might be impacted by unmeasured confounding factors, just as in any epidemiological investigation. In addition, the identification of depression relied on self-reporting by participants instead of clinical machine examination or more conclusive diagnostic approaches, which could introduce recall bias. Finally, previous depressive episodes or the presence of inflammatory diseases in the studied population are variables influencing depressive episodes and inflammation. Due to the limitations of the data, we were unable to measure relevant variables, which may have affected the results.

## Conclusion

In summary, we discovered a notable U-shaped relationship between increased exposure to EO and the occurrence of depression in a representative population of the United States. Furthermore, the inflammatory response may serve as a plausible biological pathway for EO-induced depression. Further prospective investigations are necessary to validate these findings.

## Data Availability

The survey data are publicly available on the internet for data researchers throughout the world (www.cdc.gov/nchs/nhanes/).

## Abbreviations

EO: Ethylene oxide
HbEO: Hemoglobin adducts of ethylene oxide
NHANES: National Health and Nutrition Examination Survey
NCHS: National Center for Health Statistics
PHQ-9: Patient Health Questionnaire
HPLC–MS/MS: High-performance liquid chromatography coupled with tandem mass spectrometry
BMI: Body mass index
PIR: Household poverty-to-income ratio
RCS: Restricted cubic spline
